# The safety of a novel early mobilization protocol conducted by ICU physicians: a prospective observational study

**DOI:** 10.1186/s40560-018-0281-0

**Published:** 2018-02-20

**Authors:** Keibun Liu, Takayuki Ogura, Kunihiko Takahashi, Mitsunobu Nakamura, Hiroaki Ohtake, Kenji Fujiduka, Emi Abe, Hitoshi Oosaki, Dai Miyazaki, Hiroyuki Suzuki, Mitsuaki Nishikimi, Alan Kawarai Lefor, Takashi Mato

**Affiliations:** 1 Advanced Medical Emergency Department and Critical Care Center, Japan Red Cross Maebashi Hospital, 3-21-36 Asahi-cho, Maebashi, Gunma 371-0014 Japan; 2 Department of Biostatistics, Nagoya University Graduate School of Medicine, Tsurumai-cho 64, Syowa-ku, Nagoya, Aichi 466-8560 Japan; 3 Department of Rehabilitation Medicine, Japan Red Cross Maebashi Hospital, 3-21-36 Asahi-cho, Maebashi, Gunma 371-0014 Japan; 4 Department of Nursing, Intensive Care Unit, Japan Red Cross Maebashi Hospital, 3-21-36 Asahi-cho, Maebashi, Gunma 371-0014 Japan; 5 Department of Surgery, Jichi Medical University, 3311-1 Yakushiji, Shimotsukeshi, Tochigi, 329-0498 Japan; 6 Department of Emergency Medicine, Jichi Medical University, 3311-1 Yakushiji, Shimotsukeshi, Tochigi, 329-0498 Japan

**Keywords:** Early mobilization, Protocol, Safety, ICU physicians, Medical devices, Acute phase

## Abstract

**Background:**

There are numerous barriers to early mobilization (EM) in a resource-limited intensive care unit (ICU) without a specialized team or an EM culture, regarding patient stability while critically ill or in the presence of medical devices. We hypothesized that ICU physicians can overcome these barriers. The aim of this study was to investigate the safety of EM according to the Maebashi EM protocol conducted by ICU physicians.

**Methods:**

This was a single-center prospective observational study. All consecutive patients with an unplanned emergency admission were included in this study, according to the exclusion criteria. The observation period was from June 2015 to June 2016. Data regarding adverse events, medical devices in place during rehabilitation, protocol adherence, and rehabilitation outcomes were collected. The primary outcome was safety.

**Results:**

A total of 232 consecutively enrolled patients underwent 587 rehabilitation sessions. Thirteen adverse events occurred (2.2%; 95% confidence interval, 1.2–3.8%) and no specific treatment was needed. There were no instances of dislodgement or obstruction of medical devices, tubes, or lines. The incidence of adverse events associated with mechanical ventilation or extracorporeal membrane oxygenation (ECMO) was 2.4 and 3.6%, respectively. Of 587 sessions, 387 (66%) sessions were performed at the active rehabilitation level, including sitting out of the bed, active transfer to a chair, standing, marching, and ambulating. ICU physicians attended over 95% of these active rehabilitation sessions. Of all patients, 143 (62%) got out of bed within 2 days (median 1.2 days; interquartile range 0.1–2.0).

**Conclusions:**

EM according to the Maebashi EM protocol conducted by ICU physicians, without a specialized team or EM culture, was performed at a level of safety similar to previous studies performed by specialized teams, even with medical devices in place, including mechanical ventilation or ECMO. Protocolized EM led by ICU physicians can be initiated in the acute phase of critical illness without serious adverse events requiring additional treatment.

**Electronic supplementary material:**

The online version of this article (10.1186/s40560-018-0281-0) contains supplementary material, which is available to authorized users.

## Background

After surviving a critical illness, many patients suffer long-term cognitive and physical dysfunction, and reduced health-related quality of life [1–6]. Several studies have shown that about half of patients cannot return to work [7, 8]. This has a major impact on patients, their families, and society. Recently, early mobilization (EM) in the intensive care unit (ICU) has been recommended to prevent or limit cognitive and physical dysfunction [9]. EM provides many benefits, such as reducing the duration of delirium, improved muscle strength, and improved quality of life [10–14]. EM can decrease both ICU and hospital length of stay [10, 13, 15], increase ventilator-free days [11], and improve the rate of discharge to home [11]. The safety, feasibility, and effectiveness of EM have been extensively reported [11, 14, 16–18]. EM has become an evidence-based practice and should be incorporated in daily practice, starting in the early phase of critical illness in the ICU [19].

However, many studies of EM, showing the successive introduction of active mobilization in ICU, were conducted at universities and hospitals in Europe and the USA which have specialized EM personnel or teams and have developed an “EM culture” in the ICU [10–18]. There are barriers to conducting EM as routine practice in the ICU, where there are few specialized EM teams and EM is not yet routine practice. The lack of a formalized mobilization program, an environment without a priority for EM, lack of available medical or personnel, the need for a specialized team, and the lack of specialists to lead the effort have been reported as barriers to implement EM [19–23]. In Japan, many hospitals are faced with these barriers [22] and there are few reports of the introduction of active EM in the ICU. It is unknown whether EM can be safely initiated and performed in Japanese ICUs in hospitals without a specialized team or an EM culture.

Referring to existing EM protocols reported in prior studies [9, 11, 12, 14–16, 19, 24–26], we developed a novel EM program, the Maebashi EM protocol, which is conducted at the bedside by ICU physicians in our closed mixed ICU. The EM protocol is a novel system, with the ICU physician as the key person to manage EM safely. The purpose of this study is to investigate whether EM according to this ICU physician-conducted protocol can be safely performed in the ICU without a specialized EM team or an EM culture, even though the patients have undergone the placement of a variety of medical devices. Another aspect of the study is to evaluate whether EM led by an ICU physician can be initiated in the acute phase of critical illness.

## Methods

### Study design

This is a single center prospective observational study. The study was approved by the ethics committee of the Japan Red Cross Maebashi Hospital and followed the STROBE guidelines [27]. This study is registered in UMIN (ID: 00002289).

### Hospital setting

Japan Red Cross Maebashi Hospital is a tertiary care hospital (560-bed general hospital in Gunma prefecture, Japan), with a 12-bed closed-mixed ICU. Admission sources to the ICU are the emergency room and hospital wards. Admissions from the emergency room to the ICU are all due to unplanned emergency critical illness and from the hospital wards are due to planned post-operative or unplanned emergency conditions which develop in the ward. ICU physicians and nurses (the nurse-to-patient ratio is 1:2) are present in the ICU, but there are no physical therapists assigned to the ICU. ICU physician staff includes one ICU consultant (attending physician), three ICU fellows, and one junior resident. None of them are specialized in rehabilitation. The fellows and resident treat patients with an appropriate level of supervision by the ICU consultant.

### Patients

All consecutive patients 18 years of age or older with an unplanned admission to the ICU from June 2015 to June 2016 are included in this study. Patients with planned post-operative, acute cardiovascular, acute cerebrovascular disease, progressive neuromuscular disease, post cardiopulmonary arrest syndrome, or a condition limiting mobilization such as an unstable pelvic fracture were excluded. Informed consent was obtained from all enrolled patients, if they were conscious, or from family members if the patient was unconscious.

### The Maebashi early mobilization protocol

An EM working group was formed to discuss how to promote EM in the ICU. The working group included two ICU physicians and three ICU nurses, who are not specialized in EM, and one rehabilitation doctor and three physical therapists, who are not also specialized in EM and do not usually engage in ICU rehabilitation. Non-specialized means that they are not trained specifically to provide rehabilitation for critically ill ICU patients with ICU-related medical devices in place, in the acute phase of critical illness. The EM working group confirmed that the staff who participated in this study and provided rehabilitation had no specific training in rehabilitation before this study. The EM working group sent ICU physicians, ICU nurses, and physical therapists a questionnaire to investigate barriers to care in the ICU [see Additional file 1]. After summarizing the results of the questionnaire, this group reviewed available literature and created the Maebashi EM protocol in May 2015 [see Additional file 2], which was specifically developed to be used in this ICU. A 1-month training period was used to teach ICU physicians, ICU nurses, and physical therapists how to conduct the protocol. The details of conducting rehabilitation sessions were taught by using charts at each rehabilitation level made by the EM working group [see Additional file 3].

**Fig. 1:**
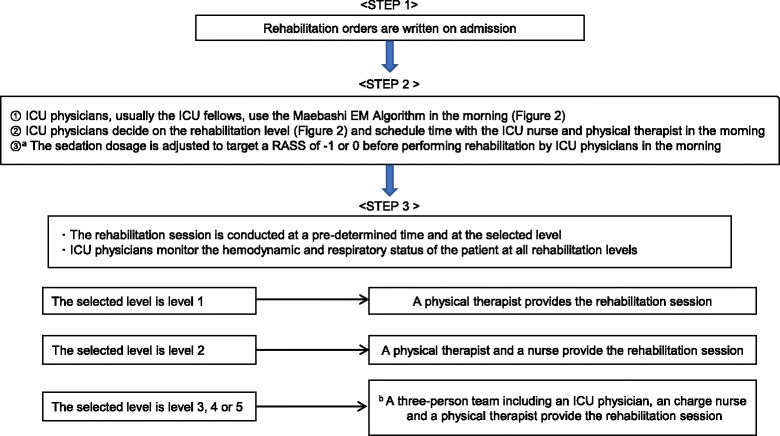
The Maebashi early mobilization protocol. *ICU* intensive care unit, *EM* early mobilization, *RASS* Richmond agitation sedation scale. **a** The sedation adjusting strategy depends on ICU physicians without any sedation protocol. **b** If the physical therapist cannot attend the session, the team is still three people and includes a physician, a charge nurse, and another ICU nurse

**Fig. 2:**
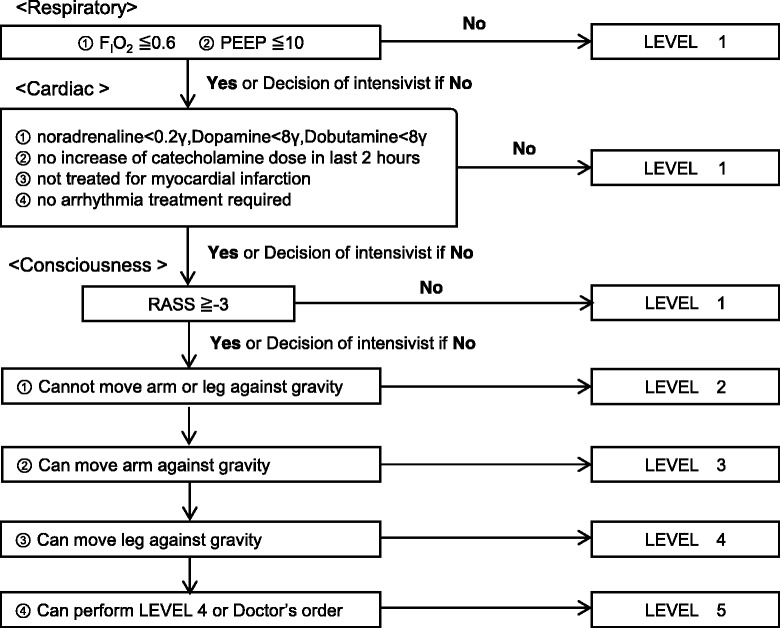
The Maebashi Early Mobilization Algorithm: a flow chart, *PEEP* positive end-expiratory pressure, *RASS* Richmond agitation sedation scale. This is the Maebashi early mobilization protocol algorithm. ICU physicians have to decide the mobilization level according to the algorithm every day. The contents of the mobilization level is as follows: level 1: no mobilization, bed exercise such as passive range of motion and passive transfer to chair; level 2: sitting position in bed, including using cycling ergometer and active range of motion; level 3: sitting on edge of bed; level 4: active transfer to chair; level 5: standing, stepping in place, and ambulating

The Maebashi EM protocol consists of three steps and includes five levels of rehabilitation. The details of the steps and rehabilitation levels are shown in Figs. 1 and 2. The five levels of rehabilitation are as follows: (1) no mobilization or bed exercise (2) sitting position in bed, including using a cycling ergometer and active range of motion (3) sitting on the edge of the bed, (4) active transfer to the chair, and (5) standing, stepping in place, or ambulating. Although all patients are supposed to receive one rehabilitation session each day for 20 min, the actual rehabilitation period was determined by ICU physicians based on the patient’s clinical condition. The role of the ICU physician during active rehabilitation sessions was to monitor the hemodynamic and respiratory status of the patient and to maintain vigilance over the central venous catheter, ECMO cannula, or endotracheal tube. Discontinuation criteria are defined as follows: a fall to the knees or ground, tachycardia (> 130/min) or bradycardia(< 40/min), hypertension (systolic blood pressure > 180 mmHg), hypotension (systolic blood pressure < 80 mmHg), symptomatic orthostatic hypotension, arrhythmias except a pre-existing arrhythmia, myocardial infarction-associated symptoms, desaturation (peripheral capillary oxygen saturation < 88%), abnormal respiratory rate (> 40/min or < 5/min), asynchrony with mechanical ventilation, patient’s intolerance to request to stop rehabilitation, cardiopulmonary arrest, bleeding, unexpected/inadvertent removal of medical devices (an endotracheal tube, feeding tube, chest tube, abdominal drain, urinary catheter, arterial catheter, peripheral or central venous catheter, or hemodialysis catheter.)

If an event meets any of the discontinuation criteria, the patient stops the rehabilitation session and rests. If the patient recovers, the rehabilitation session is reinitiated at the same rehabilitation level or at a lower level based on the judgment of the ICU physician. If the patient could not recover or requests to discontinue the session, it is stopped immediately and counted as an adverse event. A serious adverse event was defined as an adverse event requiring additional treatment.

### Study outcomes

The primary outcome was the safety of EM conducted according to the Maebashi EM protocol. The safety objective was the incidence rate of adverse events in all rehabilitation sessions. Rehabilitation levels and the types of adverse events were recorded and reviewed to evaluate the safety of EM. Medical devices in place during rehabilitation sessions were also reviewed to investigate any possible relationship between adverse events and equipment.

Secondary outcomes include the number of days to first rehabilitation and the number of days to progress to higher rehabilitation levels. Other outcomes of rehabilitation sessions, including the percentage of patients who got out of bed, standing, or ambulating, were also collected. The active rehabilitation level was defined to be sitting out of the bed, active transfer to a chair, standing, marching, and ambulating.

### Data collection

Baseline medical characteristics of all enrolled patients were collected on admission, and during the course of the ICU stay, including age, gender, body mass index (BMI), the ability to ambulate prior to ICU admission, admission source, reason for admission to the ICU, Acute Physiology and Chronic Health Evaluation II (APACHE II) score, Sequential Organ Failure Assessment (SOFA) score on admission, the need for mechanical ventilation, Extracorporeal Membrane Oxygenation (ECMO), continuous analgesia, continuous sedation, vasopressors, corticosteroids, neuromuscular blocking agents, or dialysis. Other information such as ICU length of stay, hospital length of stay, mechanical ventilation periods, ability to ambulate at hospital discharge, mortality, and where the patient went after leaving the hospital were recorded at the time of discharge.

Rehabilitation information was recorded immediately after the session on that day, including the highest level of rehabilitation which continued for at least 5 min, medical devices in place during the session, the site of each medical device, any event which met the criteria for discontinuing the session, whether the session was conducted according to protocol, and if there were any protocol violations.

There are no missing data in this study. All data were collected prospectively and sent to personnel uninvolved with the EM working group. After data collection, rehabilitation outcomes were summarized, and the relationships between adverse events and the rehabilitation level, or adverse events and medical devices were examined.

### Subgroup analysis and sensitivity analysis

To reduce the influence of the patients who had mild critical illnesses and were discharged from the ICU early, a subgroup analysis was conducted as a post hoc study, focusing on rehabilitation outcomes. Data from patients who stayed in the ICU for more than 72 h were analyzed. In the main analysis, rehabilitation information, including the number of rehabilitation sessions performed at each level, the number and rate of adverse events, the percentage of patients who got out of bed, standing or ambulating in the ICU, number of days to first rehabilitation, and number of days to progress to higher levels of rehabilitation, were summarized.

### Statistical analysis

Distributed continuous variables without a normal distribution are presented as median and interquartile range (IQR). Categorical data are summarized using numbers or percentages. The Wilcoxon rank sum test was used for comparing continuous variables, and the chi-squared test was used for categorical data. Same statistic measures were used for the sub-group analysis.

In this study, the primary outcome was set as the incidence rate of adverse events among all rehabilitation sessions, following previous study designs of safety [10, 16, 25]. The sample size was calculated at a 95% confidence level. The assumed rate of adverse events was set at 3.0% (0.03), and the expected confidence interval was 0.03, based on the rate of adverse events of the prior studies [12, 14, 16, 17, 25, 26, 28]. According to a power calculation, a total sample size of 497 rehabilitation sessions are needed to assess the safety of EM according to the Maebashi EM protocol driven by ICU physicians. To enhance the internal validity of the safety, the incidence rate of adverse events for active rehabilitation levels (levels 3, 4, and 5) and non-active rehabilitation levels (levels 1 and 2) were compared by the chi-squared test, as done in a prior study [16]. All statistical analyses were conducted using EZR (Saitama Medical Center, Jichi Medical University, Saitama, Japan), which is a graphical user interface for R (The R Foundation for Statistical Computing, Vienna, Austria) [29]. Statistical tests were two sided and statistical significance was defined as *P* < 0.05.

## Results

### Baseline patient characteristics

During the observation period from June 2015 to June 2016, 839 patients were admitted to the ICU. The details of study patient recruitment are shown in Additional file 4 [see Additional file 4]. A total of 232 patients were enrolled in this study. Table 1 shows the baseline characteristics of enrolled patients. The median age was 69.0 years (IQR 55.8–80.0 years) and 156/232 (67%) patients were male. Of 232 patients, 181 (78%) were admitted from the emergency department, 72 (31%) underwent mechanical ventilation and six (2.6%) received ECMO. The APACHE II and SOFA scores on admission were 16 (IQR 10–22) and 4 (IQR 2–7) and the average length of ICU stay and duration of mechanical ventilation were 1.8 and 2.1 days, respectively.

**Table 1:** Baseline patient characteristics (*n* = 232)

Variable	Values median [IQR] or number (%)
Age (years), median [IQR]	69.0 [55.8–80.0]
Gender (male), *n* (%)	156 (67%)
BMI (kg/m^2^), median [IQR]	21.1 [18.8–24.2]
Ambulatory prior to admission, *n* (%)	208 (90%)
Admitted from	
Emergency room, *n* (%)	181 (78%)
Hospital ward, *n* (%)	51 (22%)
ICU admission diagnosis	
Sepsis, *n* (%)	92 (40%)
Gastrointestinal, *n* (%)	49 (21%)
Respiratory failure, *n* (%)	29 (13%)
Trauma, *n* (%)	28 (12%)
Drug abuse, *n* (%)	12 (5%)
Others, *n* (%)	22 (9%)
APACHE II score, median [IQR]	16 [10–22]
SOFA on admission, median [IQR]	4 [2–7]
Patients undergoing mechanical ventilation, *n* (%)	72 (31%)
Patients receiving ECMO, *n* (%)	6 (2.6%)
Patients receiving continuous analgesia (opiates), *n* (%)	117 (50%)
Patients receiving continuous sedation, *n* (%)	82 (35%)
Patients receiving vasopressors, *n* (%)	87 (38%)
Patients receiving steroids, *n* (%)	39 (17%)
Patients receiving neuromuscular blocking agents, *n* (%)	2 (0.90%)
Patients receiving dialysis, *n* (%)	34 (15%)
ICU length of stay (days), median [IQR]	1.8 [1.2–3.7]
Mechanical ventilation period (days), median [IQR]	2.1 [0.9–4.2]
Hospital length of stay (days), median [IQR]	16.9 [9.3–36.1]
Ambulatory at discharge, *n* (%)	184 (79%)
In-hospital mortality, *n* (%)	11 (4.7%)
Discharged to	
Home, *n* (%)	138 (60%)
Another hospital or rehabilitation center, *n* (%)	72 (31%)
Nursing home, *n* (%)	11 (4.7%)

Data in table are presented as the median with the interquartile range or as a number with percentage in total patients

*BMI* body mass index, *IQR* interquartile range, *ICU* intensive care unit, *APACHE* Acute Physiology and Chronic Health Evaluation, *SOFA* Sequential Organ Failure Assessment, *ECMO* extracorporeal membrane oxygenation

### Safety

#### Rehabilitation sessions and adverse events

A total of 587 rehabilitation sessions were conducted for 232 patients. The median number of rehabilitation sessions per patient was 1 (range 0–55 sessions). The relationship between rehabilitation sessions and adverse events is summarized in Table 2. During 587 rehabilitation sessions, 13 adverse events occurred. The primary outcome, the incidence rate of adverse events among all rehabilitation sessions was 2.2% (95% confidence interval [CI] 1.2–3.8%). The adverse events included seven episodes of patient intolerance, necessitating discontinuing the rehabilitation session, and six episodes of orthostatic hypotension with symptoms (Table 3). Thirteen adverse events occurred in 10 patients; 2 patients experienced adverse events several times. One patient had three adverse events as intolerance at same rehabilitation level (level 5), and the other patient had two adverse events as orthostatic hypotension with symptoms at different levels (level 2 and 3). There was no significant difference between the incidence rate in active rehabilitation, (levels 3 to 5, 387 sessions, 11 adverse events, 2.8%; 95% confidence interval [CI] 1.4–5.0%) and the incidence rate for non-active rehabilitation, (levels 1 and 2, 200 sessions, 2 adverse events, 1.0%; 95% confidence interval [CI] 1.0–3.6%), (*P* = 0.15). There were no serious adverse events requiring additional treatment, such as cardiopulmonary resuscitation, an increase in vasopressor dose, the fraction of inspired oxygen, or the need for additional analgesia (Table 3).

**Table 2:** Rehabilitation sessions and adverse events

	Total number of sessions performed	Adverse events, *n* (%)	Total number of patients (*n* = 232)^a^
Rehabilitation level			
Level 1, *n*	154	1 (0.60%)	73
Level 2			
Total, *n*	46	1 (2.2%)	26
Ergometer, *n*	10	0 (0%)	4
Level 3, *n*	169	7 (4.1%)	74
Level 4, *n*	54	0 (0%)	18
Level 5			
Total, *n*	164	4 (2.4%)	83
Standing or marching at bedside, *n*	103	4 (3.9%)	42
Ambulating in the ICU, *n*	61	0 (0%)	49
Active rehabilitation, *n*	387	11 (2.8%)	143
Total Rehabilitation sessions, n	587	13 (2.2%)	

Data are presented as number (%)

*ICU* intensive care unit

^a^This demonstrates the number of the patients who experienced the each rehabilitation levels

**Table 3:** Type and frequency of adverse events

	Adverse events (*n* = 13)	Event rate per 1000 rehabilitation sessions
Event		
Patient intolerance^a^	7 (54%)	12
Symptomatic orthostatic hypotension	6 (46%)	10
Fall to knees or ground	0 (0%)	0
Asynchrony with mechanical ventilation	0 (0%)	0
Tachycardia or bradycardia	0 (0%)	0
Arrhythmia	0 (0%)	0
Myocardial infraction associated symptom	0 (0%)	0
Tachypnea or bradypnea	0 (0%)	0
Desaturation	0 (0%)	0
Cardiopulmonary arrest	0 (0%)	0
Bleeding	0 (0%)	0
Inadvertent removal of medical devices	0 (0%)	0

Data are presented as number of occurrences with percentage

A total of 587 rehabilitation sessions were performed during the study period

^a^Patients’ intolerance includes five episodes of extreme exhaustion and two episodes of exacerbation of abdominal pain in patients diagnosed with acute pancreatitis. There is no scale for exhaustion or pain

### Relationship between medical devices and adverse events

Medical devices in place during the rehabilitation sessions are summarized in Table 4 Nearly all rehabilitation sessions were performed with peripheral venous catheters (99%), arterial lines (98%), and urinary bladder catheters (94%) in place. Other medical devices, such as chest or abdominal drains, or central venous catheters, were also in place during rehabilitation. Additional file 5 shows the details of rehabilitation sessions and adverse events related to mechanical ventilation and ECMO. Of 587 rehabilitation sessions, 293 sessions (50%) were performed while the patient was undergoing mechanical ventilation and 110 sessions (19%) were performed with ECMO devices in place. The incidence rate of adverse events in patients undergoing mechanical ventilation was 2.4% and with ECMO was 3.6%. There were no adverse events directly related to medical devices, such as inadvertent removal.

**Table 4:** Relation between medical devices and adverse events**.**

	Total number of sessions performed	Adverse events, *n* (%)
Medical devices in place during the session		
Peripheral venous catheter, *n*	582	13 (2.2%)
Arterial line		
Total, *n*	574	13 (2.3%)
Radial, *n*	568	13 (2.3%)
Femoral, *n*	6	0 (0%)
Central venous catheter		
Total, *n*	167	8 (4.8%)
Jugular, *n*	112	5 (4.5%)
Subclavian, *n*	18	0 (0%)
Femoral, *n*	37	3 (8.1%)
Hemodialysis catheter		
Total, *n*	105	1 (1.0%)
Jugular, *n*	96	1 (1.0%)
Femoral, *n*	11	0 (0%)
Mechanical ventilator, *n*	293	7 (2.4%)
Endotracheal tube, *n*	183	5 (2.7%)
Tracheostomy tube, *n*	127	3 (2.4%)
ECMO cannula, *n*		
Total, *n*	110	4 (3.6%)
Jugular, *n*	110	4 (3.6%)
Femoral, *n*	110	4 (3.6%)
Feeding tube, *n*	419	10 (2.4%)
Urinary catheter, *n*	550	12 (2.2%)
Chest tube, *n*	83	3 (3.6%)
Abdominal drain, *n*	112	2 (1.8%)
Total rehabilitation sessions	587	13 (2.2%)

Data are presented as number (%)

*ECMO* extracorporeal membrane oxygenation

### Compliance with the Maebashi EM protocol and participating staff at each level

**Fig. 3:**
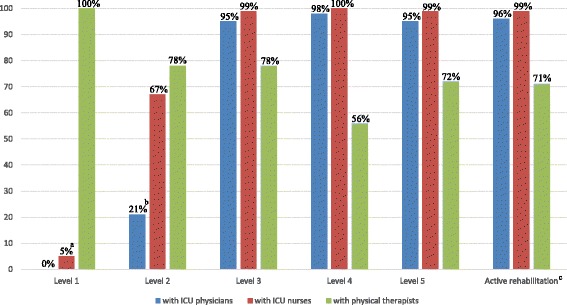
The percentage of sessions where personnel were involved at each rehabilitation session level**.** *ICU* intensive care unit. **a** The 5% means that nurses participated in the passive transfer to chair. **b** The 21% means that ICU physicians participated in the ergometer with ECMO devices to monitor ECMO cannula. **c** Active rehabilitation level includes levels 3 to 5

Protocol compliance was reviewed, and there were no violations, such as rehabilitation sessions which were not conducted according to the Maebashi EM protocol. All rehabilitation sessions were conducted strictly according to the written protocol. ICU physicians attended 96% of active rehabilitation sessions, ICU nurses attended 99%, and physical therapists attended 71% (Fig. 3). During all sessions which ICU physicians did not attend, especially at levels 1 or 2, ICU physicians were present near the rehabilitation site in the ICU and monitored the hemodynamic and respiratory status of all patients.

### Rehabilitation outcomes

Rehabilitation outcomes during the study period are summarized in Table 5. The median number of days to the first protocolized rehabilitation session was 0.7 (IQR 0.0–0.9). A total of 62% of patients (*n* = 143) got out of bed during their ICU stay, and the median time to first getting out of bed was 1.2 (IQR 0.1–2.0) days.

**Table 5:** Outcomes of protocolized rehabilitation

	All study patients (*n* = 232)^a^	Subgroup in the ICU ≧ 72 h (*n* = 71)
Variable	Values median [IQR] or number (%)	Values median [IQR] or number (%)
Patients who could get out of bed, *n* (%)	143 (62%)	58 (82%)
Patients who could stand during ICU stay, *n* (%)	82 (35%)	31 (45%)
Patients who could ambulate during ICU stay, *n* (%)	49 (21%)	12 (17%)
Days to first rehabilitation session (days), median [IQR]^a^	0.7 [0.0–0.9]	1.0 [0.8–2.0]
Days to first out of bed (days), median [IQR]^a^	1.2 [0.1–2.0]	2.0 [1.4–3.6]
Days to first standing (days), median [IQR]^a^	1.2 [0.8–2.1]	2.8 [1.7–4.9]
Days to first ambulating (days), median [IQR]^a^	1.0 [0.7–1.7]	2.3 [1.2–2.9]

Data in table are presented as the median with the interquartile range or as a number with percentage in total patients

*IQR* interquartile range, *ICU* intensive care unit

^a^Days counted from the time of ICU admission

### Subgroup analysis

There were 71 patients who stayed in the ICU for more than 72 h, including 41 (59%) who underwent mechanical ventilation, with mean APACHE II scores of 23 (IQR 18–28), and the length of ICU stay or mechanical ventilation were 5.2 days (IQR 3.8–7.8) or 3.9 days (IQR 2.1–6.8), respectively [see Additional file 6]. The rehabilitation outcomes in this subgroup are summarized in Table 5. The rehabilitation sessions began within 1 day (median 1.0 days; IQR 0.8–2.0 days), and 82% (58) of patients could get out of bed within 2 days (median 2.0 days; IQR 1.4–3.6 days).

## Discussion

This is the first study from Japan to demonstrate the safety of EM in the ICU. There are few studies focusing on the direct involvement of ICU physicians in EM. In this study, two important clinical outcomes were observed. First, EM conducted by ICU physicians according to a protocol, without a specialized EM team or an EM culture, results in a rate of adverse events similar to that reported in previous studies [12, 14, 16, 17, 25, 26, 28]. There were no adverse events related to in situ medical devices. Second, EM conducted by ICU physicians can be initiated in the acute phase of critical illness without serious adverse events requiring additional treatment or resuscitation. These results suggest that ICU physician-conducted EM is safely performed in an environment where EM was not routine practice, available resources are limited, and there is no specialized EM team, EM specialists, or an EM culture.

EM conducted by ICU physicians according to a protocol is performed at a safety level comparable to that reported in prior studies, in the absence of a specialized EM team, and in patients with a variety of medical devices in place. This study identified 13 adverse events (2.2%; 95% CI 1.2–3.8%), which did not require specific treatment and occurred at the incidence rate of adverse events similar to previous studies conducted in institutions with a specialized EM team [12, 14, 16, 17, 25, 30]. EM conducted in patients undergoing mechanical ventilation or ECMO, which have been considered barriers to active rehabilitation or relatively high risk [25, 31], was also safely performed with a low incidence rate of adverse events (2.4 and 3.6%, respectively). In an ICU without a specialized team or an EM culture, the lack of specially trained personnel to manage safety during EM and provide leadership among multidisciplinary ICU staff [10, 32], limited numbers of personnel [18, 24], the presence of medical devices [25, 31] have all been considered major barriers to initiate EM in the ICU [19–21]. A questionnaire was given to members of the ICU staff to identify the barriers in the ICU, which revealed similar barriers for the initiation of EM [see Additional file 1]. ICU physicians are trained to lead and cooperate with other staff, to manage clinical problems and to deal with problems associated with medical devices [33]. The leadership, cooperation, and medical management skills of ICU physicians are essential to initiate EM in such an environment. This skill set matches some of the perceived difficulties of initiating EM in the ICU, making the ICU physician an ideal person to lead such an effort. The Maebashi EM protocol adopted a simple algorithm and simple rehabilitation content based on previous studies which were successively introduced in the ICU [see Additional file 2] and did not utilize specialized rehabilitation equipment such as electrical muscle stimulation. Due to the simplicity of the protocol, ICU physicians who are not specialized in rehabilitation can initiate and make clinical decisions regarding EM. In this study, ICU physicians were directly involved in over 95% of active rehabilitation sessions and safely provide protocolized EM.

EM, guided by an ICU physician conducted protocol, can be initiated in the acute phase of critical illness without serious adverse events requiring additional treatment. Prior studies showed that respiratory and hemodynamic instability, which are familiar problems in acute illness, are commonly perceived as barriers by some staff, such as nurses or physical therapists [19–21]. Another study pointed out that mobilization in the acute phase of critical illness may be difficult because of severity [34], though early initiation of rehabilitation is recommended to improve patient outcomes because of rapid muscle atrophy within 24 to 48 h after ICU admission [11, 35, 36].

Although the involvement of specialists or a specialized team have been recommended to promote EM in the acute phase of critical illness [37–40], many hospitals in Japan do not have a specialized team to conduct this therapy. In these situations, the ICU physician can play an important role. As part of their training, ICU physicians develop the requisite skills to manage respiratory and hemodynamic problems in acutely ill patients [33, 41]. Some studies suggest that the involvement of ICU physicians may reduce complications and potentially enhance the safety of ICU procedures [42–44]. If adverse events associated with critical illness occur, ICU physicians can cope with events immediately and appropriately. There were no serious adverse events requiring additional treatment or resuscitation in this study. ICU physicians play an important role as a safety net in the conduct of EM in the acute phase of critical illness.

In this study, the length of ICU stay (1.8 days) and the duration of mechanical ventilation (2.1 days) are shorter than previous studies [11, 15]. Patients who received mechanical ventilation represent 31% of enrolled patients. It may seem natural that many patients could get out of bed early in their ICU stay, since their critical illness was not so severe. Therefore, we conducted a subgroup analysis, focusing on patients who stayed in the ICU for more than 72 h (Table 5, [see Additional file 6]). The average length of ICU-stay (5.2 days) and duration of mechanical ventilation (3.9 days) were comparable to a prior study (5.9 and 3.4 days respectively) [11]. Patients in the sub-group were more severely ill (median APACHE II score 23) and underwent mechanical ventilation more frequently (59%) compared to all enrolled patients. This subgroup analysis also confirmed the safety of the EM protocol (adverse events in 2.7%, and no serious adverse events) and showed positive rehabilitation outcomes (82% of the subgroup-patients could get out of bed within 2 days).

This study has several acknowledged limitations. First, patients with certain diseases were excluded. Due to relatively stringent patient selection criteria, severe critical ill patients were excluded and relatively mild severe patients were included, and the results of this study may not be generally applicable. Patients with diseases excluded from this study were immobilized for a long period and were thought not to be suitable for active rehabilitation strategies in the acute phase of critical illness, especially within 1 day. Other protocols or strategies might be necessary for patients with these excluded conditions. Patients with post-operative scheduled admission to the ICU were also excluded, because almost all of them stayed in the ICU for a very short period and were usually discharged early the next morning before receiving the rehabilitation sessions.

There may be unrecognized confounding factors associated with adverse events. For example, data regarding agitation, delirium, the rate of ICU-acquired weakness, or muscle atrophy were not collected in this study. Although the clinical workload of ICU physicians and nurses was increased due to this protocol, any relationship between an increase in daily work and adverse events was not examined.

Third, the statistical method to count adverse events and rehabilitation sessions was a repeated measurement which could influence the results. We used the method described in a previous study and a recent systematic review with a meta-analysis to enhance the comparability of safety among studies [10, 16, 25, 45]. It is important to take repeated measurement data into account when the sample size is calculated, which is a limitation of this study. The total number of patients was included to evaluate the rate of the adverse events per patient at each rehabilitation level, in addition to the rate of adverse events per session. This analysis allows consideration of the rate of adverse events without the influence of individual patient characteristics.

Fourth, this is a single-center observational study without a comparison group, which could introduce bias, limiting the ability to generalize these results to other hospitals. Further observation and verification, focusing on the other factors associated with the safety of EM or the short- and long-term effects of EM according to the protocol on clinical outcomes, is necessary to investigate the external validity and the utility of the Maebashi EM protocol.

## Conclusion

EM, performed according to the Maebashi EM protocol conducted by ICU physicians, without EM specialists, an EM specialized team or an EM culture, was performed with a safety level similar to that reported in previous studies which were conducted with a specialized team, even though patients had a variety of medical devices in place. Protocolized EM led by ICU physicians can be initiated in the acute phase of critical illness without serious adverse events requiring additional treatment.

## Additional files

Additional file 1: Barriers described by ICU physicians, ICU nurses, and physical therapists. (DOCX 15 kb)

Additional file 2: References for details of the Maebashi early mobilization protocol. (DOCX 17 kb)

Additional file 3: Example of each rehabilitation level. (DOCX 4361 kb)

Additional file 4: Details of exclusion criteria and a flow diagram of recruitment. *ICU* intensive care unit. There were no missing data. (PPTX 43 kb)

Additional file 5: Rehabilitation sessions and adverse events—mechanical ventilation and ECMO. (DOCX 15 kb)

Additional file 6: Characteristics of patients who stayed in the ICU more than 72 h. (DOCX 15 kb)

## Abbreviation

APACHE: Acute Physiology and Chronic Health Evaluation II
BMI: Body mass index
CI: Confidence interval
ECMO: Extracorporeal membrane oxygenation
EM: Early mobilization
ICU: Intensive care unit
IQR: Interquartile range
SOFA: Sequential Organ Failure Assessment
