# Supplementary material for: The safety of a novel early mobilization protocol conducted by ICU physicians: a prospective observational study
Source: J Intensive Care. 2018 Feb 20;6:10. doi: 10.1186/s40560-018-0281-0 (PMC5819168; doi:10.1186/s40560-018-0281-0)
Supplement: Supplementary file 1 — Barriers described by ICU physicians, ICU nurses, and physical therapists. (DOCX 15 kb) [file 40560_2018_281_MOESM1_ESM.docx]

**Additional File 1**

**Barriers described by ICU physicians, ICU nurses and physical therapists.**

|  | ICU physicians | ICU nurses | Physical therapists |
| --- | --- | --- | --- |
| List | Lack of knowledge and experience about rehabilitation  Lack of program and strategy  Lack of EM culture in ICU  Lack of multidisciplinary communication and cooperation  Limited stuff, time constrains | Lack of priority for early mobilization  Lack of program and strategy  Lack of EM culture in ICU  Lack of knowledge and experience about rehabilitation  Lack of multidisciplinary communication and cooperation  Lack of resources, such as personnel, equipment, costs  Lack of the leadership  Lack of conference round  ICU-related medical devices  Hemodynamic or respiratory instability of the patients  Risk management during rehabilitation  Poor management of the time schedule among medical staffs  Deep sedation  Excessive daily workload  Inadequate staff training | Lack of priority for early mobilization  Lack of program and strategy  Lack of EM culture in ICU  Lack of multidisciplinary communication and cooperation  Lack of share of the patient condition  Lack of personnel resources  ICU-related medical devices  Hemodynamic or respiratory instability of the patients  Poor management of the time schedule　among medical staffs  Severity of the patients  Risk management during rehabilitation  Ambiguous bed rest level |
| Frequently mentioned barriers | Lack of knowledge and experience  Lack of program and strategy | Hemodynamic or respiratory instability of the patients  ICU-related medical devices  Lack of program and strategy  Poor management of the time schedule among stuff  Lack of the leadership  Risk management during rehabilitation  Lack of multidisciplinary communication and cooperation | Hemodynamic or respiratory instability of the patients  ICU-related medical devices  Lack of program and strategy  Severity of the patients  Risk management during rehabilitation  Lack of multidisciplinary communication and cooperation |

*ICU* intensive care unit, *EM* early mobilization

This questionnaire survey was conducted during the protocol development phase.

The common barrier among ICU physicians, ICU nurses, and physical therapist were the lack of program and strategy.

Many ICU nurses and physical therapists mainly mentioned that they could not provide rehabilitation only by themselves because of the fear of the instability in the acute phase of critical illness and the ICU related devices.

To overcome these barriers, we conducted the early mobilization protocol conducted by ICU physicians with ICU nurses and physical therapists.
