# Supplementary material for: The safety of a novel early mobilization protocol conducted by ICU physicians: a prospective observational study
Source: J Intensive Care. 2018 Feb 20;6:10. doi: 10.1186/s40560-018-0281-0 (PMC5819168; doi:10.1186/s40560-018-0281-0)
Supplement: Supplementary file 2 — References for details of the Maebashi early mobilization protocol. (DOCX 17 kb) [file 40560_2018_281_MOESM2_ESM.docx]

**Additional File 2**

**References for details of the Maebashi Early Mobilization Protocol**

| Contents of the protocol |  |  | Reference number |
| --- | --- | --- | --- |
| <STEP 1> automatic rehabilitation order |  |  | 1, 2, 3 |
| <Algorithm> Respiratory |  |  | 4, 5 |
| <Algorithm> Cardiac; dosage of the catecholamine |  |  | 6 |
| <Algorithm> Cardiac: other items |  |  | 1, 4, 7 |
| <Algorithm> consciousness |  |  | 1 |
| <Algorithm> rehabilitation contents at each level |  |  | 1, 8 |
| Adverse events |  |  | 1, 7, 9, 10, 11 |
| Protocol exclusion criteria: |  |  | 1, 4, 7, 9 |

Reference number

1. Morris PE, Goad A, Thompson C, et al. Early intensive care unit mobility therapy in the treatment of acute respiratory failure. Crit Care Med 2008;36:2238-2243.
2. Cameron S, Ball I, Cepinskas G, Choong K, Doherty TJ, Ellis CG, et al. Early mobilization in the critical care unit: A review of adult and pediatric literature. J Crit Care 2015;30(4):664-72.
3. Jolley SE, Regan-Baggs J, Dickson RP, Hough CL. Medical intensive care unit clinician attitudes and perceived barriers towards early mobilization of critically ill patients: a cross-sectional survey study. BMC Anesthesiol 2014;14:84.
4. Hodgson CL, Stiller K, Needham DM, et al. Expert consensus and recommendations on safety criteria for active mobilization of mechanically ventilated critically ill adults. Crit Care 2014;18:658.
5. Baily P, Thomsen GE, Spuhler VJ, Jewkes J, Bezdjian L, Veale K, et al. Early activity is feasible and safe in respiratory failure patients. Crit Care Med 2007;35(1):139-45.
6. Burtin C, Clerckx B, Robbeets C, et al. Early exercise in critically ill patients enhances short-term functional recovery. Crit Care Med 2009;37:2499-2505.
7. Pohlman MC, Schweickert WD, Pohlman AS, et al. Feasibility of physical and occupational therapy beginning from initiation of mechanical ventilation. Crit Care Med. 2010;38:2089-2094.
8. Collings N, Cusack R. A repeated measures, randomized cross-over trial, comparing the acute exercise response between passive and active sitting in critically ill patients. BMC Anesthesiol. 2015;15:1.
9. Schweickert WD, Pohlman MC, Pohlman AS, et al. Early physical and occupational therapy in mechanically ventilated, critically ill patients: a randomized controlled trial. Lancet 2009;373:1874-182.
10. Sricharoenchai T, Parker AM, Zanni JM, Nelliot A, Dinglas VD, Needham DM. Safety of physical therapy interventions in critically ill patients: a single center prospective evaluation of 1110 intensive care unit admissions. J Crit Care 2014;29:395-400.
11. Lee H, Ko YJ, Yang JH, Park CM, Jeon K, Park YH, et al. Safety profile and feasibility of early physical therapy and mobility for critically ill patients in the medical intensive care unit: Beginning experiences in Korea. J Crit Care 2015;30(4):573-7.
