# Supplementary material for: The safety of a novel early mobilization protocol conducted by ICU physicians: a prospective observational study
Source: J Intensive Care. 2018 Feb 20;6:10. doi: 10.1186/s40560-018-0281-0 (PMC5819168; doi:10.1186/s40560-018-0281-0)
Supplement: Supplementary file 3 — Example of each rehabilitation level. (DOCX 4361 kb) [file 40560_2018_281_MOESM3_ESM.docx]

**Additional File 3**

**Example of each rehabilitation level**

These charts were used in the training among medical staffs including ICU physicians, ICU nurses, and physical therapists during the training period.

[level 1]

No mobilization

Bed exercise such as passive range of motion were provided by a physical therapist. Passive transfer to chair is conducted by a physical therapist and ICU nurses.


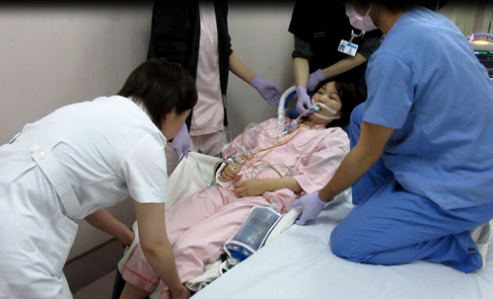
 Passive transfer to chair

[Level 2]

Sitting position in bed were performed by a ICU nurse.

Active range of motion were performed by a physical therapist.

Cycling ergometer were performed by a physical therapist and a ICU nurse. If there was the ECMO device with the patient, the ICU physician monitor the ECMO device and cannula.


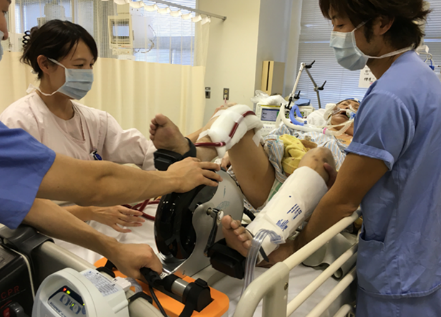
 Cycling ergometer

[Level 3]

Sitting on edge of bed is performed by a ICU physician, a nurse, and a physical therapist basically.


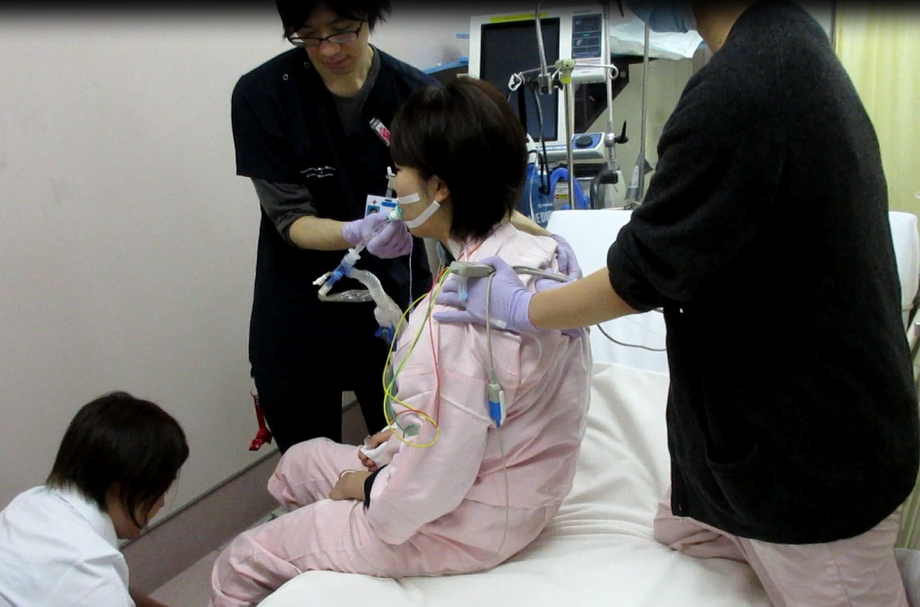
 Sitting on edge of bed

[Level 4]

Active transfer to chair is performed by a ICU physician, a nurse, and a physical therapist basically.


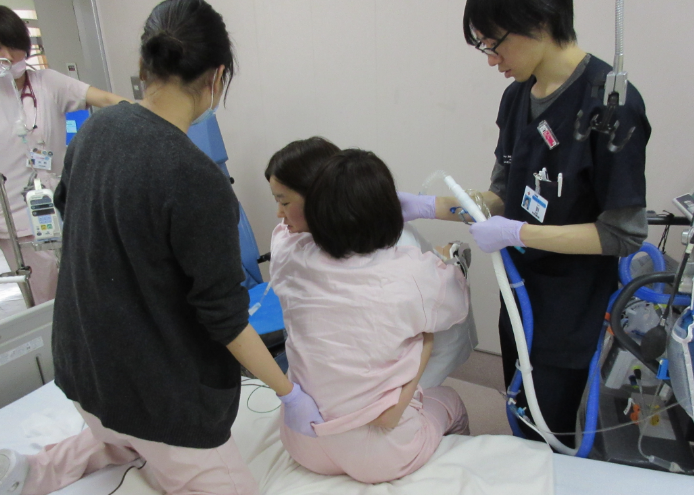

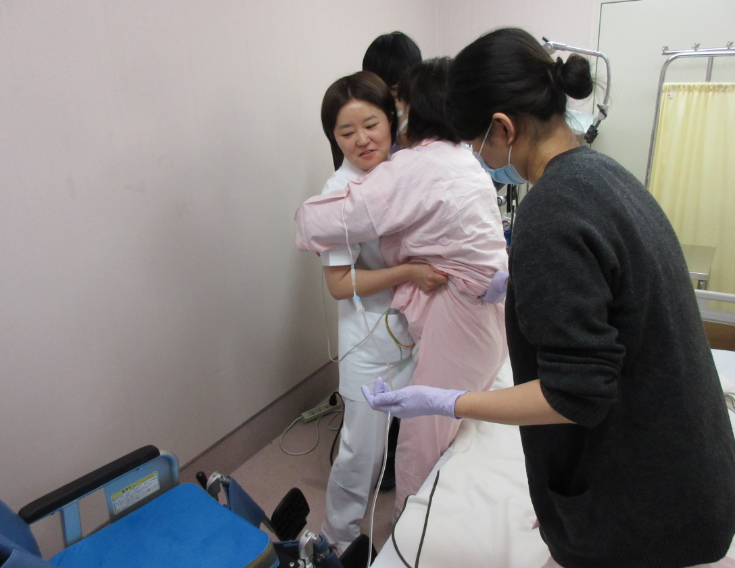

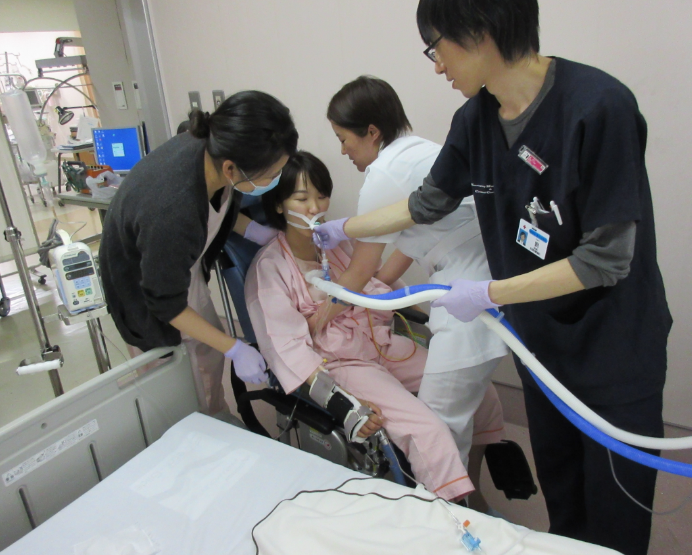


Active transfer to chair

[Level 5]

Standing, stepping in place is performed by a ICU physician, a nurse, and a physical therapist basically.


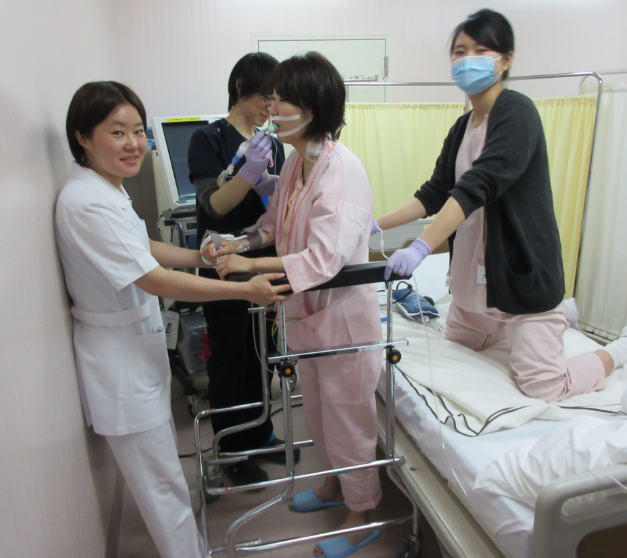
 Standing, stepping in place

Ambulating is performed by a ICU physician, a nurse, and a physical therapist basically. If another nurse has time, she may help and participate in the ambulating.


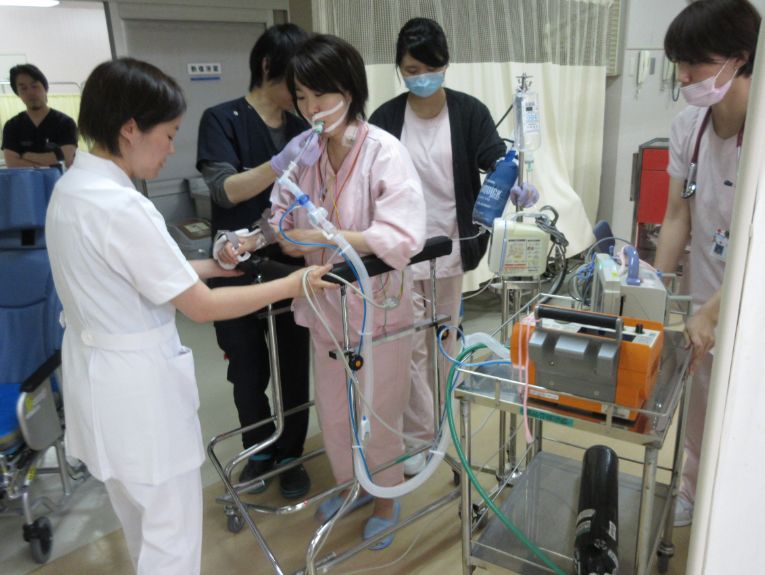
 Ambulating
