# Supplementary material for: The safety of a novel early mobilization protocol conducted by ICU physicians: a prospective observational study
Source: J Intensive Care. 2018 Feb 20;6:10. doi: 10.1186/s40560-018-0281-0 (PMC5819168; doi:10.1186/s40560-018-0281-0)
Supplement: Supplementary file 4 — Details of exclusion criteria and a flow diagram of recruitment. ICU intensive care unit. There were no missing data. (PPTX 43 kb) [file 40560_2018_281_MOESM4_ESM.pptx]

## Slide 1
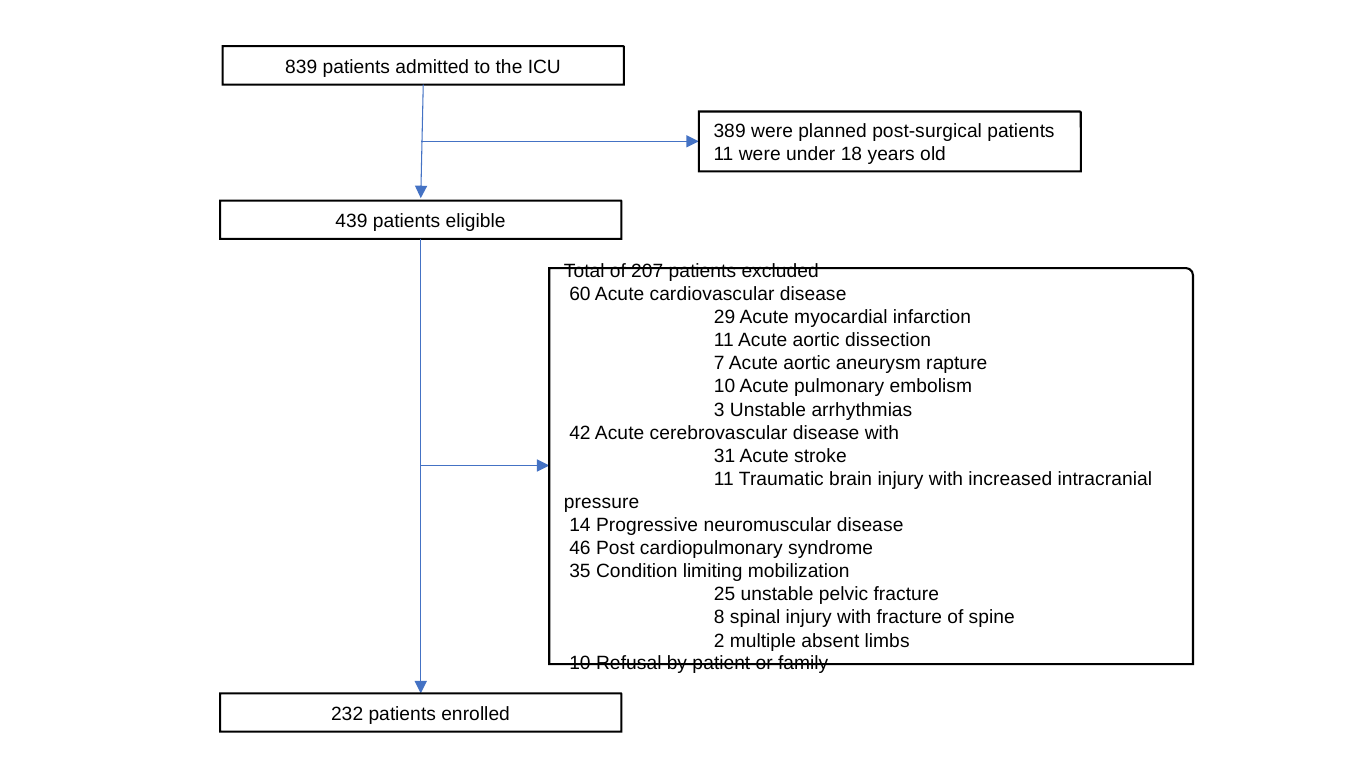

839 patients admitted to the ICU
389 were planned post-surgical patients
11 were under 18 years old
439 patients eligible
Total of 207 patients excluded
 60 Acute cardiovascular disease
	29 Acute myocardial infarction
	11 Acute aortic dissection
	7 Acute aortic aneurysm rapture
	10 Acute pulmonary embolism
	3 Unstable arrhythmias
 42 Acute cerebrovascular disease with
	31 Acute stroke
	11 Traumatic brain injury with increased intracranial pressure
 14 Progressive neuromuscular disease
 46 Post cardiopulmonary syndrome
 35 Condition limiting mobilization
	25 unstable pelvic fracture
	8 spinal injury with fracture of spine
	2 multiple absent limbs
 10 Refusal by patient or family
232 patients enrolled
