# Supplementary material for: The safety of a novel early mobilization protocol conducted by ICU physicians: a prospective observational study
Source: J Intensive Care. 2018 Feb 20;6:10. doi: 10.1186/s40560-018-0281-0 (PMC5819168; doi:10.1186/s40560-018-0281-0)
Supplement: Supplementary file 5 — Rehabilitation sessions and adverse events—mechanical ventilation and ECMO. (DOCX 15 kb) [file 40560_2018_281_MOESM5_ESM.docx]

**Additional File 5**

**Rehabilitation sessions and Adverse Events – Mechanical ventilation and ECMO**

|  |  |  |  |  |
| --- | --- | --- | --- | --- |
|  | Rehabilitation sessions with mechanical ventilation (n=293) | | Rehabilitation sessions with ECMO (n=110) | |
| Variable | Total number of sessions performed | Adverse events, n (%) | Total number of sessions performed | Adverse events, n (%) |
| ***Rehabilitation level*** |  |  |  |  |
| Level 1, n | 94 | 0 (0%) | 34 | 0 (0%) |
| Level 2 |  |  |  |  |
| total, n | 35 | 1 (2.9%) | 17 | 1 (5.9%) |
| ergometer, n | 10 | 0 (0%) | 9 | 0 |
| Level 3, n | 93 | 3 (3.2%) | 29 | 1 (3.4%) |
| Level 4, n | 15 | 0 (0%) | 0 | 0 (0%) |
| Level 5, n | 56 | 3 (5.4%) | 30 | 2 (6.7%) |
| Active rehabilitation, n^a^ | 164 | 6(3.7%) | 59 | 3(5.1%) |
| Total Rehabilitation sessions, n | 293 | 7 (2.4%) ^b^ | 110 | 4 (3.6%) ^c^ |
|  |  |  |  |  |

Data in table are presented as a number with percentage in total patients.

*ECMO* extracorporeal membrane oxygenation, *ICU* intensive care unit

^a^ Active rehabilitation level include level 3 to 5.

^b^ There was no significant difference between adverse events rate with mechanical ventilation and those with other medical equipment (294 sessions, 6 adverse events, 2.0%): 2.4% vs 2.0% P=0.77

^c^ There was no significant difference between adverse events rate with ECMO and those with other medical equipment (477 sessions, 9 adverse events, 1.9%): 3.6% vs 1.9% P=0.2
