# Supplementary material for: The safety of a novel early mobilization protocol conducted by ICU physicians: a prospective observational study
Source: J Intensive Care. 2018 Feb 20;6:10. doi: 10.1186/s40560-018-0281-0 (PMC5819168; doi:10.1186/s40560-018-0281-0)
Supplement: Supplementary file 6 — Characteristics of patients who stayed in the ICU more than 72 h. (DOCX 15 kb) [file 40560_2018_281_MOESM6_ESM.docx]

**Additional File 6**

**Characteristics of patients who stayed in the ICU more than 72 hours**

|  | Patients ≧ 72 hours (n=71) |
| --- | --- |
| Variable | Values Median [IQR] or Number (%) |
| Age (years), median [IQR] | 72.0 [63.0-80.0] |
| Gender (male), n (%) | 43 (61%) |
| BMI (kg/m^2^), median [IQR] | 21.3 [18.8-24.2] |
| Ambulatory prior to admission, n (%) | 61 (86%) |
| APACHE II score, median [IQR] | 23 [18-28] |
| SOFA at admission, median [IQR] | 8 [5-10] |
| Patients receiving mechanical ventilation, n (%) | 41 (59%) |
| Patients receiving ECMO, n (%) | 6 (8.1%) |
| Patients receiving continuous analgesia (opiates), n (%) | 51 (72%) |
| Patients receiving sedation, n (%) | 51 (72%) |
| Patients receiving vasopressor, n (%) | 49 (69%) |
| Patients receiving steroid, n (%) | 22 (31%) |
| Patients receiving neuromuscular blocking agent, n (%) | 0 (0%) |
| Patients receiving dialysis, n (%) | 24 (34%) |
| ICU Length of stay (days), median [IQR] | 5.2 [3.8-7.8] |
| Hospital Length of stay (days), median [IQR] | 36.3 [17.4-61.1] |
| Mechanical ventilator periods (days), median [IQR] | 3.9 [2.1-6.8] |
| Ambulatory at hospital discharge, n (%) | 46 (65%) |
| In-hospital mortality, n (%) | 10 (14.1%) |
| Discharged to |  |
| Home, n (%) | 25 (35%) |
| Another hospital or rehabilitation center, n (%) | 32 (45%) |
| Nursing home, n (%) | 4 (5.6%) |
|  |  |
| ***Rehabilitation level*** | Total number of rehabilitation sessions |
| Level 1, n | 113 |
| Level 2, n | 32 |
| Level 3, n | 126 |
| Level 4, n | 35 |
| Level 5 |  |
| total, n | 109 |
| Standing or marching at bedside, n | 87 |
| Ambulating in the ICU, n | 22 |
| Active rehabilitation, n | 270 |
| All Rehabilitation sessions, n | 415 |
| Total adverse events, n^a^ | 11 |
|  |  |

Data in the table are presented as the median with the interquartile range or as a number with percentage in total patients.

*BMI* body mass index, *IQR* interquartile range, *ICU* intensive care unit, *APACHE* Acute Physiology and Chronic Health Evaluation, *SOFA* Sequential Organ Failure Assessment, *ECMO* extracorporeal membrane oxygenation

^a^ The rate of adverse events was 2.7 % (95% confidence interval, 1.3-4.7%). These 11 adverse events included six episodes of patient’s intolerance (four were extreme exhaustion and two were exacerbation of abdominal pain with acute pancreatitis) and five episodes of orthostatic hypotension with symptoms
